# A new immune checkpoint-associated nine-gene signature for prognostic prediction of glioblastoma

**DOI:** 10.1097/MD.0000000000033150

**Published:** 2023-03-03

**Authors:** Xiao Jin, Xiang Zhao

**Affiliations:** a The Personnel Department, Dongfang Hospital Affiliated to Beijing University of Chinese Medicine, Fengtai District, Beijing, China; b Department of Neurosurgery, The First Hospital of China Medical University, Shenyang, Liaoning, China.

**Keywords:** cell death, glioma, immunotherapy, LASSO-cox regression, pyroptosis

## Abstract

Glioblastoma (GBM) is a highly malignant neurological tumor that has a poor prognosis. While pyroptosis affects cancer cell proliferation, invasion and migration, function of pyroptosis-related genes (PRGs) in GBM as well as the prognostic significance of PRGs remain obscure. By analyzing the mechanisms involved in the association between pyroptosis and GBM, our study hopes to provide new insights into the treatment of GBM. Here, 32 out of 52 PRGs were identified as the differentially expressed genes between GBM tumor versus normal tissues. And all GBM cases were assigned to 2 groups according to the expression of the differentially expressed genes using comprehensive bioinformatics analysis. The least absolute shrinkage and selection operator analysis led to the construction of a 9-gene signature, and the cancer genome atlas cohort of GBM patients were categorized into high risk and low risk subgroups. A significant increase in the survival possibility was found in low risk patients in comparison with the high risk ones. Consistently, low risk patients of a gene expression omnibus cohort displayed a markedly longer overall survival than the high risk counterparts. The risk score calculated using the gene signature was found to be an independent predictor of survival of GBM cases. Besides, we observed significant differences in the expression levels of immune checkpoints between the high risk versus low risk GBM cases, providing instructive suggestions for immunotherapy of GBM. Overall, the present study developed a new multigene signature for prognostic prediction of GBM.

## 1. Introduction

Glioblastoma (GBM) is a highly aggressive central nervous system malignant tumor with high mortality and bad prognosis.^[1]^ Worse still, GBM represents around 57% of all gliomas as well as 48% of all primary brain malignancies and its median survival is <2 years.^[2]^ The conventional strategy for treatment of the newly diagnosed GBM patients involves surgery combined with concurrent radiotherapy using temozolomide followed by adjuvant temozolomide. Alternatively, adjuvant temozolomide can be administered concurrently with cancert-treating fields, delivering low intensity alternating electric fields.^[2]^ While a number of biomarkers and gene signatures show the promising potential for prognostic prediction of GBM, there still exists a gap between research and clinical practice. Hence, it is of clinical significance to identify gene signatures for predicting the prognosis of GBM. Given that significant alterations of the neurovascular unit occur in central nervous system malignancies including GBM, some potential immunotherapies for GBM may be available.^[3]^

Pyroptosis, a type of programmed lytic cell death, exhibits characteristic features including swelling and rupture of cells, cellular content release, as well as significant pro-inflammation effects. During pyroptosis, inflammasomes perceive danger signaling and cellular events, eliciting caspase activation, Gasdermin D cleavage, and release of IL-18 and IL-1β.^[4]^ And it can suppress tumor occurrence and progression, while forming a microenvironment for providing nutrients to cancer tissues and promoting tumor growth.^[5]^ Emerging evidence shows that it functions in tumor cell proliferation, invasion, and migration, influencing cancer prognosis.^[6,7]^ For instances, pyroptosis induces apoptosis of tumor cells in digestive tract. And NLRs3, AIM24, and GSDM5 family are critically involved in pyroptosis-related signaling in digestive cancer, including esophageal cancer, gastric cancer, and colitis-associated colorectal cancer.^[8]^ Recently, a study identified a new gene signature for prognostic prediction of ovarian cancer which is related to pyroptosis.^[9]^ Meanwhile, a prognostic signature for lung adenocarcinoma was found to contain 5 pyroptosis-related genes (PRGs) including NLRP1, NLRP2, NLRP7, NOD1, and CASP6. And lncRNA KCNQ1OT1, miR-335-5p, NLRP1, and NLRP7 were shown to form a regulatory axis that may be critically implicated in lung cancer progression.^[10]^ To date, research reports on relationship between pyroptosis and GBM are still lacking.

Herein, we examined the expression profiles of PRGs using bioinformatics approaches and divided the GBM cases of the the cancer genome atlas (TCGA) or gene expression omnibus (GEO) cohort into 2 subtypes based on the risk score. Strikingly, we found a novel gene signature with a prognostic significance which was related to immune checkpoint expression in GBM. This study may facilitate further investigation on prognostic prediction and treatment of GBM.

## 2. Methods

### 2.1. Datasets

RNA sequencing and clinical data of 169 GBM cases as well as 5 normal individuals from TCGA database were downloaded (portal.gdc.cancer.gov/repository). A GEO cohort of GBM patients (GSE83300) were used to validate the risk model. Patients in this cohort underwent a shorter duration of follow up (up to 3 years) than those in the TCGA cohort.

### 2.2. Identification of the differentially expressed genes (DEGs)

As presented in Table S1, Supplemental Digital Content, http://links.lww.com/MD/I576, a total of 52 PRGs were chosen for this study.^[9,11–13]^ Out of the 52 PRGs, 32 were identified as the DEGs using the TCGA cohort (Table S2, Supplemental Digital Content, http://links.lww.com/MD/I577). Normalization of the expression data to fragment per kilobase million values was carried out prior to comparative analysis. Identification of DEGs was carried out using the package Limma at a value of *P* < .05. The DEGs were notated as below: *** *P* < .001, ** *P* < .01, and * *P* < .05. search tool for the Retrieval of Interacting Genes version 11.5 (https://cn.string-db.org/) was employed to construct a protein–protein interaction (PPI) network.

### 2.3. Generation and external validation of the novel gene signature

To determine prognostic relevance of DEGs, Cox regression analysis was conducted to investigate association of the PRGs with survival condition of GBM cases in the TCGA cohort. A cutoff *P* value was set at 0.2 to avoid omissions. Twenty survival-associated genes were screened out for subsequent studies (Table S3, Supplemental Digital Content, http://links.lww.com/MD/I578). Thereafter, least absolute shrinkage and selection operator (LASSO) analysis was carried out with the package glmnet to further screen the candidates and develop a gene model for prognostic prediction. Finally, 9 genes and the relevant coefficients were selected, and the penalty parameter (λ) was chosen based on the minimum criteria. The gene expression data were subjected to standardization and centralization by implementing R function scale, and risk score calculation was then performed as follows: $Risk\;score=\underset{i}{\overset{7}{\sum }}\;\;Xi\times Yi$ (X and Y represent the coefficient and expression level, respectively). GBM cases of the TCGA cohort were divided into low risk and high risk subgroups using the median risk score. And Kaplan–Meier analysis was carried out to comparatively analyze the overall survival (OS) between the 2 subgroups. Nine-gene signature based principal component analysis (PCA) was conducted by the R Stats Package function prcomp. Three-year receiver operating characteristic (ROC) curve analysis was performed by using the R packages survival, survminer, and time-ROC. A GEO cohort of GBM cases was used to validate the prognostic model. DEG expression level in the GEO cohort was subjected to normalization using the function scale, and the same formula as that for the TCGA cohort was used to calculate the risk score. Like the TCGA cohort, the GEO cohort of patients were assigned to the 2 different groups according to the median risk score, and the multigene signature was validated by a comparative analysis between the 2 groups. Besides, decision curve analysis (DCA) was conducted to assess the novel signature.

### 2.4. Assessment of prognostic significance of the risk score

Clinical data of GBM cases in the TCGA cohort were extracted. And we undertook the univariate and multivariable regression analyses to determine correlations of clinical characteristics with the risk score.

### 2.5. Predictive nomogram

A nomogram represents a graphical statistical tool for quantitative assessment of risk of subjects in a clinical setting integrated with several risk factors. We developed a nomogram by integrating the prognostic signatures to evaluate 1-year, 2-year, and 3-year OS of GBM cases.

### 2.6. Functional characterization of DEGs

The GBM cases were categorized into the low risk and high risk group using the median risk score. Identification of DEGs between the 2 groups was performed using the criteria of FDR < 0.05 and |log2FC| ≥ 1, and DEGs were then subjected to gene ontology and Kyoto encyclopaedia of genes and genomes analyses using package cluster Profiler.

### 2.7. Immune activity analysis

The enrichment scores of immune cell subpopulations as well as the activities of immune associated pathways were examined by performing single-sample gene set enrichment analysis with the package GSVA. Besides, potential immune checkpoints were retrieved from previous studies.

### 2.8. Statistics

**Figure 1.**
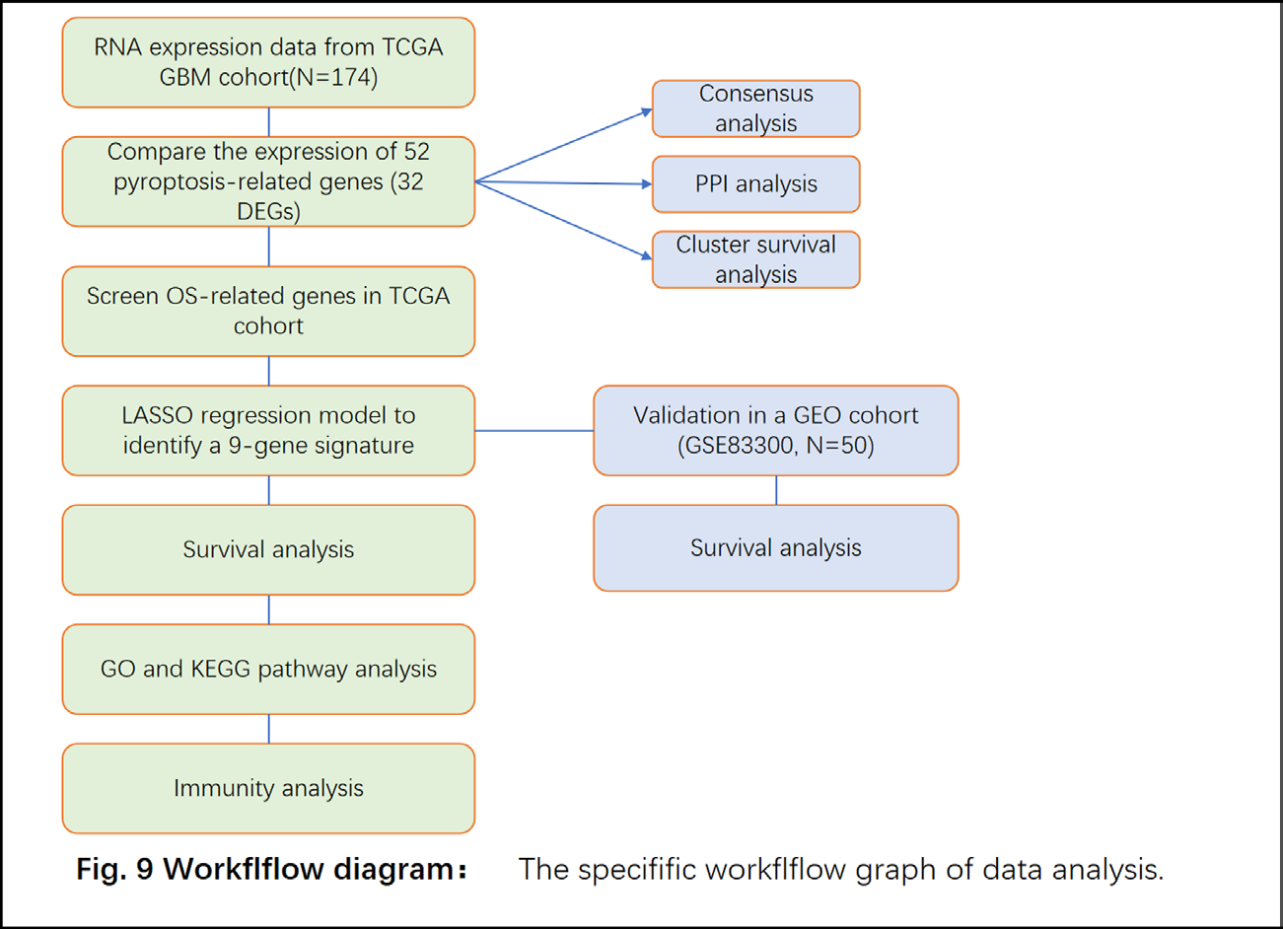
The work-flow diagram.

Comparison of the gene expression level between GBM versus normal brain tissues was made using single factor analysis of variance, and Pearson chi-square test was conducted to comparatively analyze categorical variables. Both Kaplan–Meier method and the log-rank test were employed to carry out inter group comparison of the OS. The univariate and multivariable analyses were used to assess prognostic significance of the gene signature. Significance of the model in prognostic prediction of GBM was determined by using DCA. Besides, comparison of immune cell infiltration, activation of immune associated pathways, and the expression level of immune checkpoints between the 2 subgroups was made using the Mann–Whitney test. Data were statistically analyzed with R software v4.1.0 (PMID: 36155484). Figure 1 presents the work-flow diagram in this research.

## 3. Results

### 3.1. Screening of DEGs between tumor versus normal tissues

**Figure 2.**
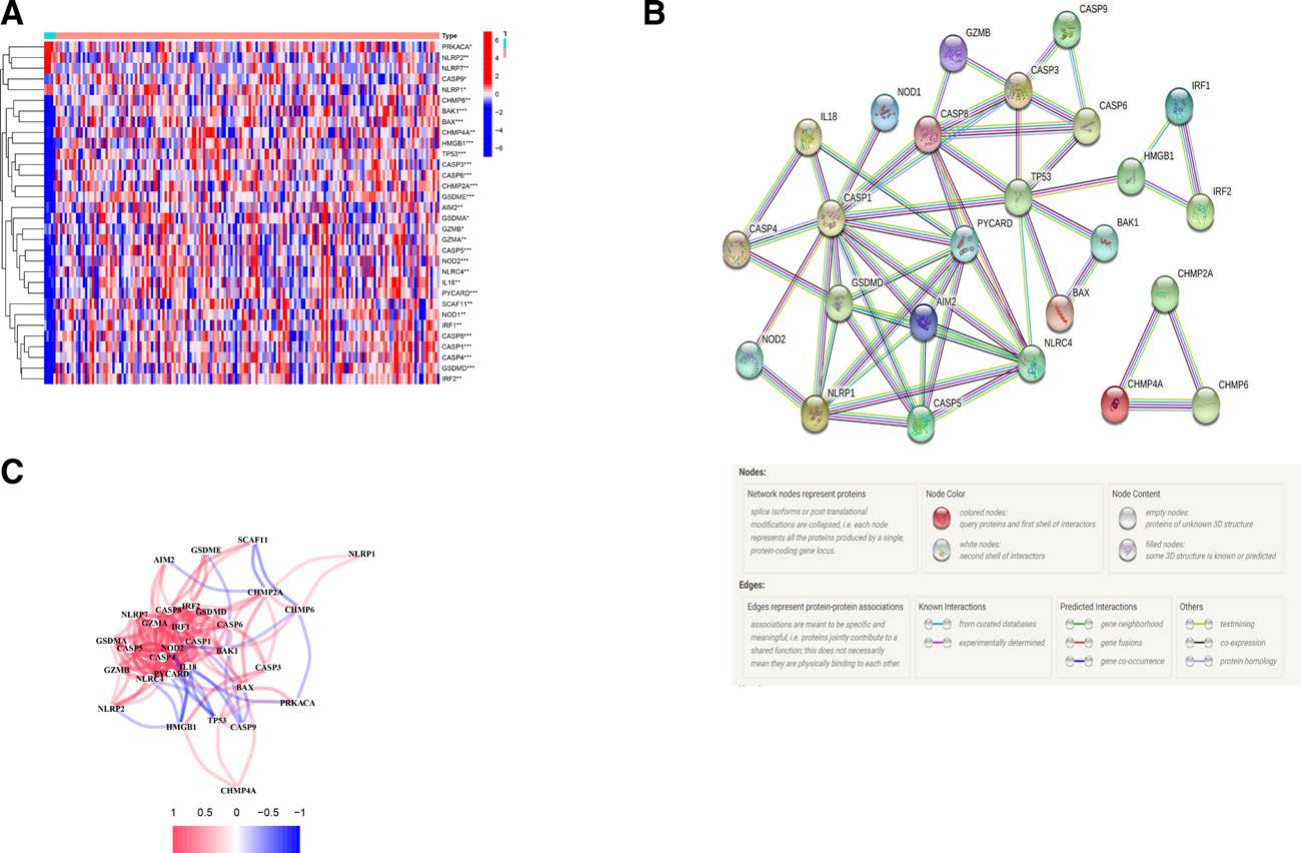
The expression and interaction analyses of 32 pyroptosis-associated DEGs. (A) A heatmap showing the relative expression levels of the DEGs between tumor versus normal tissues. Color scale: red, high expression; blue, low expression. **P < .01 and ***P < .001. (B) Interaction analysis of DEGs based on a PPI network. The highest confidence of the minimum required interaction score was 0.9. (C) Correlation analysis of DEGs. The positive and negative correlations were indicated by red and blue lines, respectively. Color depth corresponded to the correlation degree. DEGs = differentially expressed genes, PPI = protein–protein interaction.

We comparatively analyzed the expression of 52 PRGs among 169 GBM samples and 5 normal tissue samples from the TCGA. Among them, 32 were identified as the DEGs, of which 4 (*NLRP2, NLRP7, NLRP1*, and *PRKACA*) were downregulated and the remaining 28 (*CHMP6, CASP9, SCAF11, HMP4A, HMGB1, CHMP2A, IRF2, BAK1, NOD1, NLRC4, CASP8, GSDME, IRF1, BAX, CASP3, GZMB, IL18, PYCARD, gasdermin D (GSDMD), CASP6, CASP5, NOD2, TP53, AIM2, GSDMA, CASP1, CASP4*, and *GZMA*) were up-regulated in the tumor samples (Fig. 2A). We further carried out PPI analysis to investigate the interactions among the 32 DEGs. As depicted in Figure 2B, the PPI analysis identified *CASP1, GSDMD, NLRP1, AIM2, PYCARD, CASP8, CASP5, TP53*, and *CASP3* as hub genes. Moreover, we constructed a correlation network for the DEGs (Fig. 2C).

### 3.2. PRG-based tumor clustering

**Figure 3.**
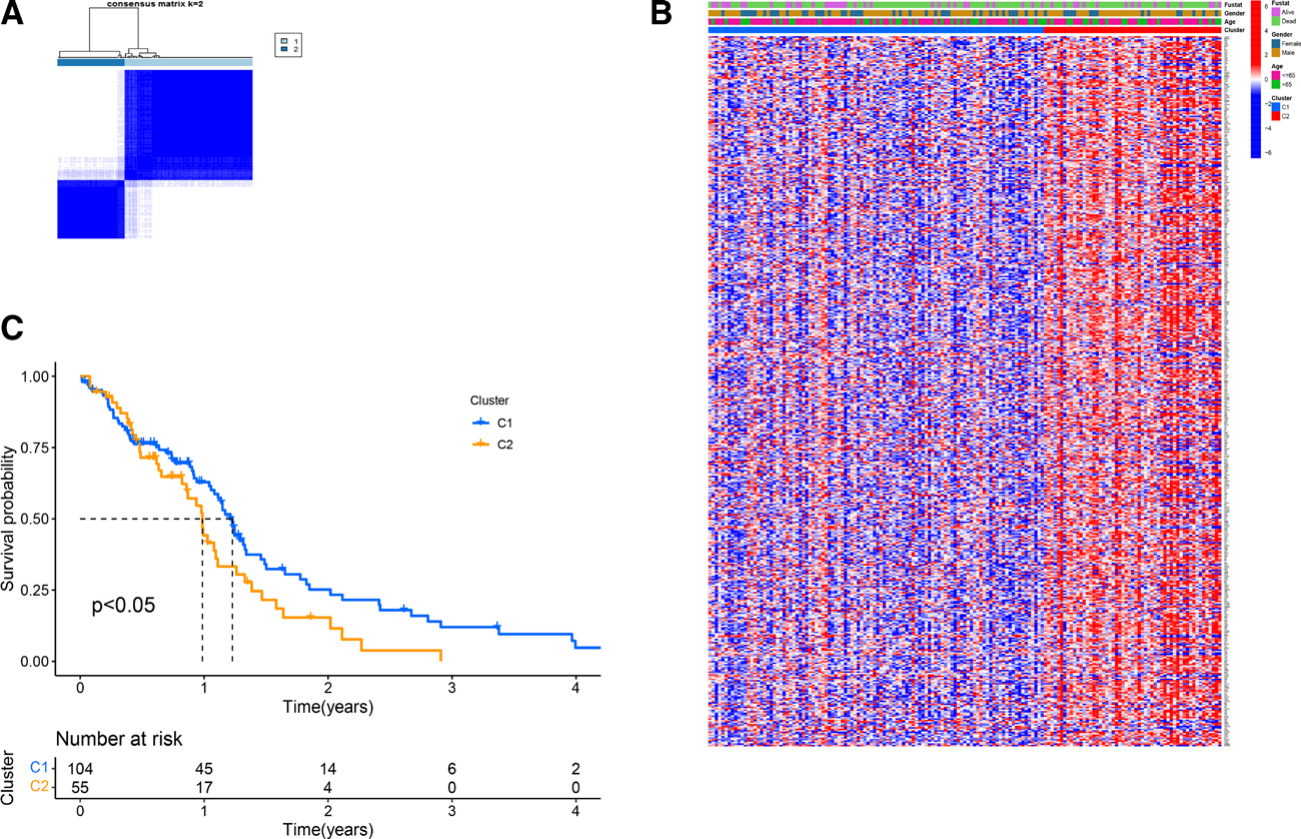
PRG-based clustering of GBM cases. (A) 169 GBM cases were assigned to 2 groups according to consensus clustering matrix for k = 2. (B) A heatmap showing clinical characteristics of the GBM cases. (C) Kaplan–Meier curves of OS. GBM = glioblastoma, OS = overall survival, PRGs = pyroptosis-related genes.

To determine the correlation of the PRG expression with GBM subgrouping, we conducted the consensus clustering analysis on the TCGA cohort of GBM cases. As depicted in Figure 3A, the highest intragroup correlations as well as low intergroup correlations were observed at the clustering variable (k) of 2, showing that all 169 GBM cases can be divided into 2 clusters according to PRG expression levels. Meanwhile, we identified 484 DEGs between the 2 clusters (Table S4, Supplemental Digital Content, http://links.lww.com/MD/I579). Notably, no marked differences in clinical characteristics including survival status, gender and age were detected between the 2 clusters (Fig. 3B). By contrast, a marked difference in the OS was detected between them (Fig. 3C).

### 3.3. Establishment of a gene model for prognostic prediction

**Figure 4.**
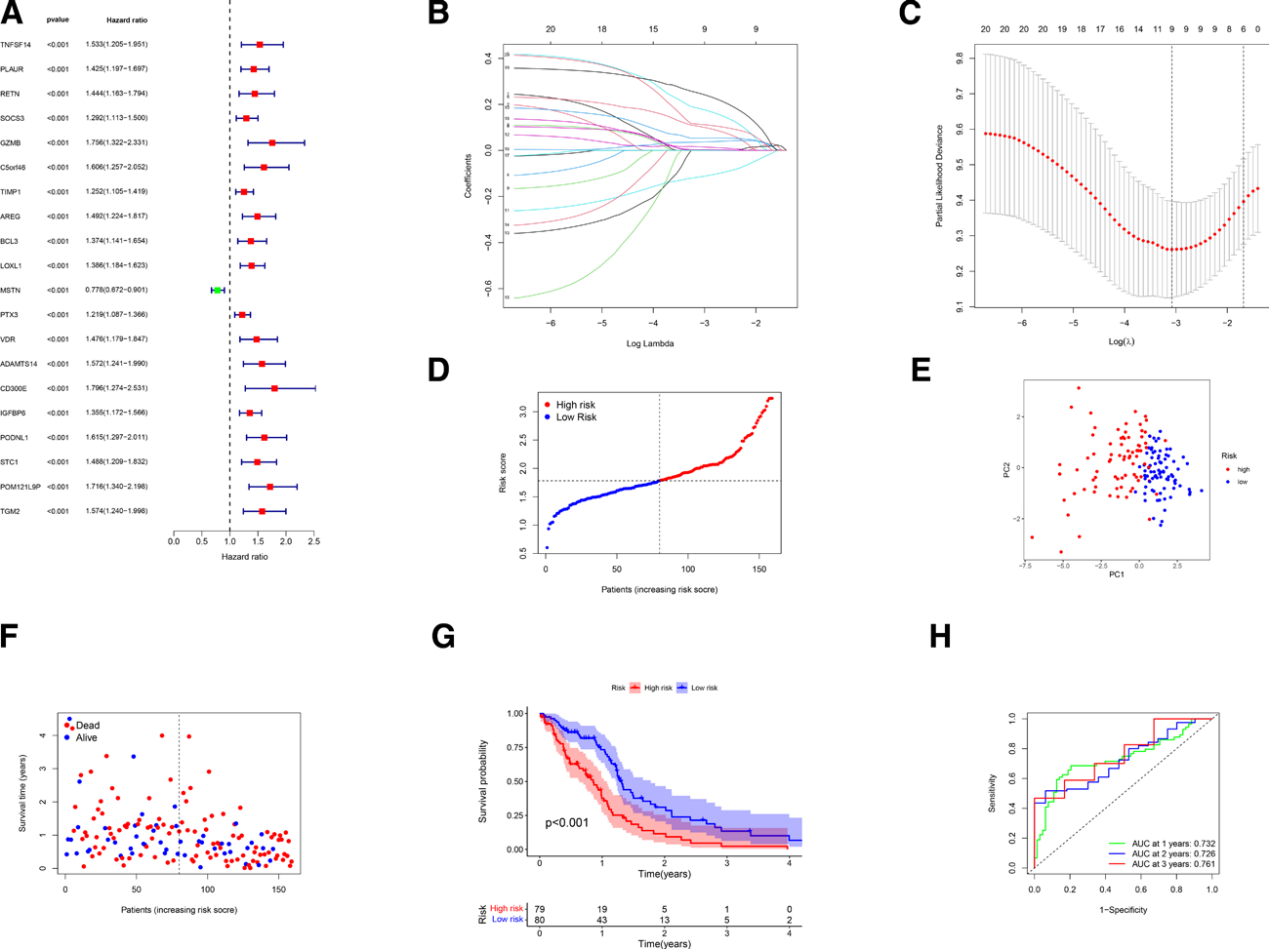
Construction of a multigene signature based on the TCGA dataset. (A) Identification of 9 survival-related PRGs by the univariate regression method (*P* < .2). (B) Construction of a 9-gene signature using LASSO regression. (C) Tuning parameter selection by cross validation in the LASSO model. (D) Risk score-based distribution of the GBM cases. (E) Score plot of PCA on the risk of the GBM patients. (F) Comparative analysis on the survival status between the 2 groups of patients. (G) Kaplan–Meier curves of the GBM cases. (H) ROC curve analysis of the gene model. GBM = glioblastoma, LASSO = least absolute shrinkage and selection operator, PCA = principal component analysis, PRGs = pyroptosis-related genes, ROC = receiver operating characteristic, TCGA = the cancer genome atlas.

We next carried out univariate Cox regression analysis on the 159 GBM patients with complete survival data to screen for survival associated genes. As illustrated in Figure 4A, 9 genes (*GZMB, AREG, LOXL1, MSTN, PTX3, IGFBP6, STC1, POM121L9P, and TGM2*) were chosen for further investigation according to the criterion of *P* < .2. Among the 9 genes, 8 (*GZMB, AREG, LOXL1, PTX3, IGFBP6, STC1, POM121L9P, and TGM2*) were found to be correlated with an increased risk (hazard ratios, HRs > 1), and the remaining 1 (*MSTN*) was identified as a protective gene (HRs < 1). LASSO analysis using the optimum λ value led to the construction of a 9-gene signature (Fig. 4B and C). We calculated the risk score by the following formula: Risk score = (0.181*GZMB exp.) + (0.104* *AREG* exp.) + (0.053*LOXL1 exp.) + (−0.115*MSTN exp.) + (0.016* *PTX3* exp.) + (0.036*IGFBP6 exp.) + (0.003*STC1 exp.) + (0.247*POM121L9P exp.) + (0.113*TGM2 exp.). One hundred-nine GBM cases were equally assigned to the high risk or low risk group based on the median risk score (Fig. 4D). Strikingly, PCA analysis revealed a clear separation of the 2 groups with a distinct risk score (Fig. 4E). In comparison with low risk cases, the high risk ones displayed more deaths as well as shortened survival time (Fig. 4F). Moreover, a marked difference in OS was observed between the 2 groups of patients (Fig. 4G). To further assess the prognostic model, we conducted ROC curve analysis. As depicted in Figure 4H, the area under the ROC curve was 0.732, 0.726, and 0.761 for 1-year, 2-year, and 3-year survival, respectively.

### 3.4. Validation of the prognostic multigene signature using a different cohort of GBM cases

**Figure 5.**
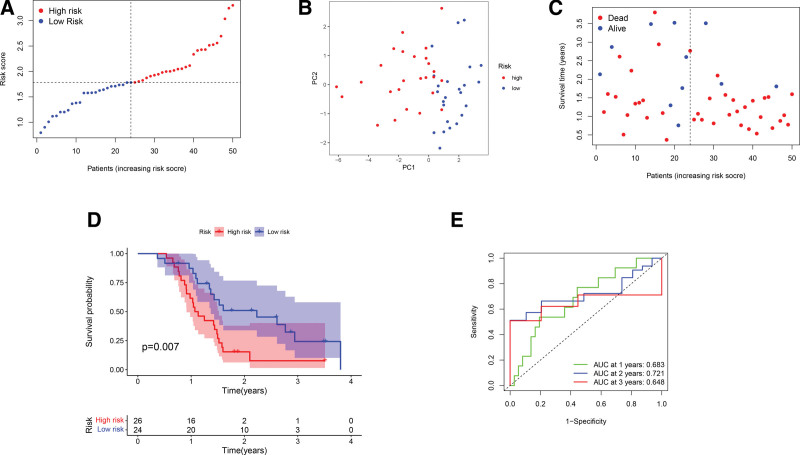
Validation of the gene model. (A) GBM cases of a GEO cohort were divided into the low risk and high risk groups. (B) Score plot of PCA on the risk of GBM patients. (C) Comparative analysis on survival status between the 2 groups. (D) Kaplan–Meier curves of the 2 groups of cases. (E) The ROC analyses of the model. GBM = glioblastoma, GEO = gene expression omnibus, PCA = principal component analysis, ROC = receiver operating characteristic.

A GEO cohort of 50 GBM cases (GSE83300) was used to conduct external validation of the prognostic signature. The expression profiles of PRGs were subjected to normalization using the “Scale” function prior to analysis. Among the 50 GEO patients, 26 and 24 were assigned to the high risk and low risk group, respectively, according to the median risk score derived from the TCGA cases (Fig. 5A). PCA analysis revealed a good separation between the 2 groups (Fig. 5B). As shown in Figure 5C, the low risk cases exhibited increased survival time as well as reduced mortality rate in comparison with the high risk cases. Besides, a marked difference in OS was identified between the 2 categories of patients (Fig. 5D). Notably, the area under the ROC curve was found to be 0.683, 0.721, and 0.648 for 1-year, 2-year, and 3-year survival, respectively, indicating that the model has good predictive efficacy for the GEO cohort (Fig. 5E).

### 3.5. The value of the signature model in prognostic prediction

**Figure 6.**
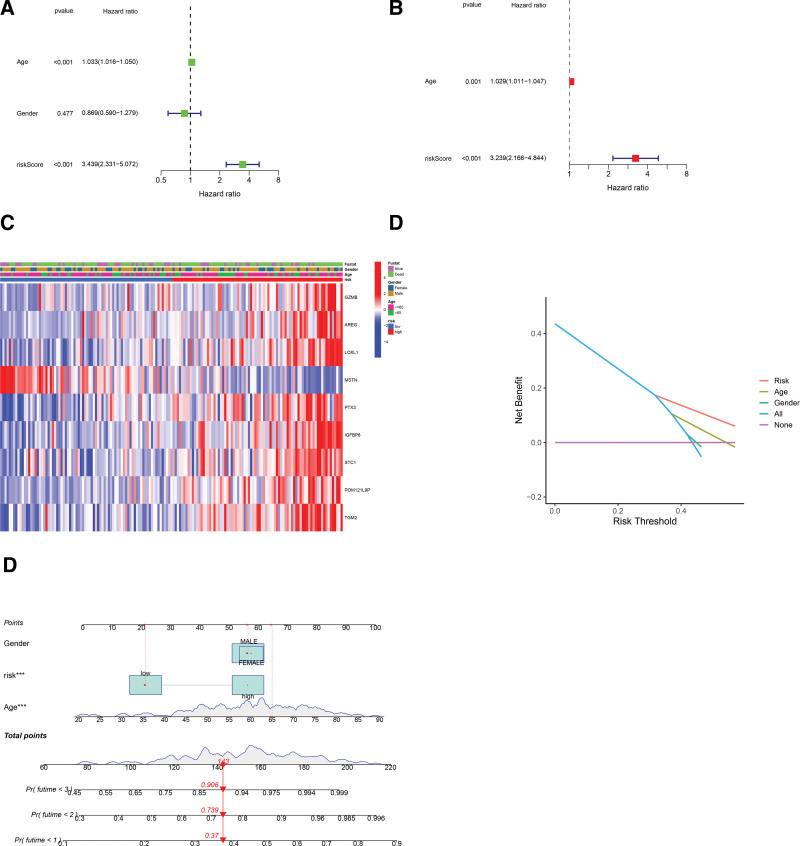
The univariate and multivariable regression analyses of the risk score. (A) The univariate analysis. (B) The multivariable analysis. (C) Heatmap showing the differences in clinicopathologic characteristics between the 2 groups of patients. *P < .05. (D) DCA analysis of the signature. (E) Nomogram combining clinical features with the signature. DCA = decision curve analysis.

To further examine the role of the signature model derived risk score in prognostic prediction, we conducted both univariate and multivariable regression analyses. As depicted in Figure 6A, the univariate regression analysis identified the risk score as an independent predictor for poor survival of GBM patients (HR = 3.439, 95% confidence interval: 2.331 − 5.072). Meanwhile, the multivariable analysis revealed that the risk score serves as a prognostic predictor of the patients (HR = 3.239, 95% confidence interval: 2.166 − 4.844) (Fig. 6B). No marked differences in clinical features were detected between the 2 groups of patients, as indicated in the heatmap (Fig. 6C). We further performed DCA analysis to determine the sensitivity and specificity of the multigene model by comparing with clinicopathological characteristics of the GBM cases. As illustrated in Figure 6D, the 9-gene signature exhibited a better performance in the prognostic prediction than the clinicopathological characteristics. Besides, the nomogram combining clinical features with the 9-gene signature produced a stable and accurate prediction of prognosis (Fig. 6E), indictive of a significant clinical value.

### 3.6. Functional characterization of the DEGs

**Figure 7.**
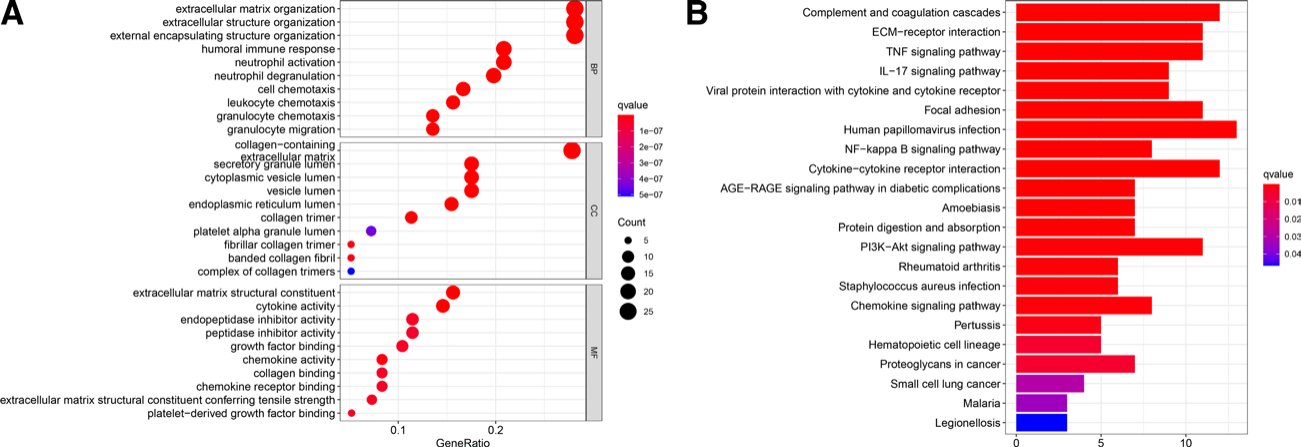
Functional characterization of the DEGs. (A) The bubble diagram showing GO annotations of the DEGs. Note that the bigger bubbles represent more enriched DEGs in the categories, while the increasing intensity of redness indicates the greater differences. The q value is an adjusted *P* value. (B) Barplot of enriched pathways. Note that the longer bars represent more enriched DEGs in the pathways, while the increasing intensity of redness indicates the greater differences. DEGs = differentially expressed genes, GO = gene ontology.

We next extracted DEGs between the 2 groups of GBM cases with package Limma and undertook functional analysis. As shown in Table S5, Supplemental Digital Content, http://links.lww.com/MD/I580, 97 DEGs were identified according to the criteria of |log2FC | ≥ 1 and FDR < 0.05; out of them, 7 were downregulated and the remaining 90 were highly expressed in the high risk patients. Furthermore, gene ontology and Kyoto encyclopaedia of genes and genomes analysis revealed a predominant enrichment of the DEGs in chemokine signaling pathway, the immune response, TNF signaling, NF − kappa B signaling, IL − 17 signaling, PI3K − Akt signaling, and AGE − RAGE signaling pathway (Fig. 7A and B).

### 3.7. Inter group comparison of the immune activities

**Figure 8.**
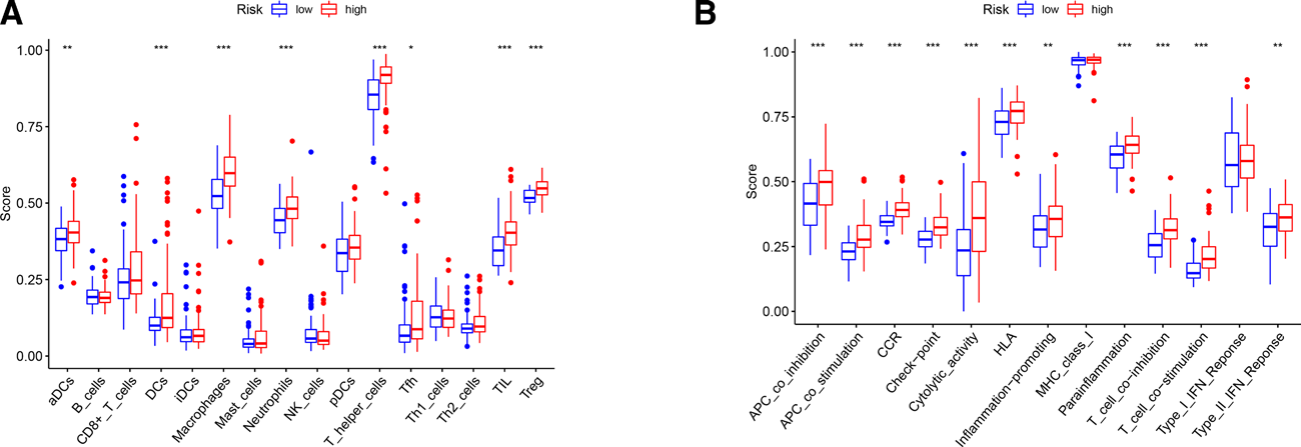
ssGSEA-based comparison of immune activities. (A and B) Comparative analysis on the enrichment scores of 16 immune cell subpopulations as well as the activities of 13 immune-associated pathways between the 2 clusters of GBM cases. *P < .05, **P < .01, and ***P < .001. GBM = glioblastoma, ssGSEA = single-sample gene set enrichment analysis.

**Figure 9.**
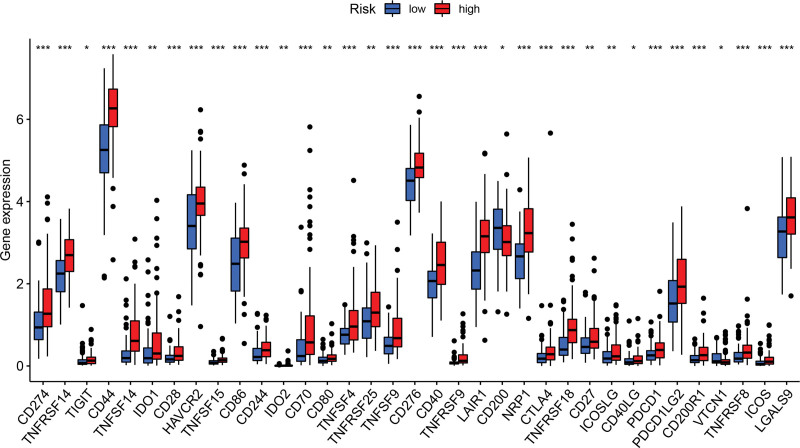
Comparative analysis on the expression level of 35 immune checkpoints between the 2 clusters of GBM cases. *P < .05, **P < .01, and ***P < .001. GBM = glioblastoma.

We further carried out single-sample gene set enrichment analysis to comparatively analyzed enrichment scores of 16 immune cell subpopulations as well as the activities of 13 immune-associated pathways between the 2 groups of GBM cases. As depicted in Figure 8A, a significant increase in the enrichment of immune cell subpopulations, including dendritic cells, macrophages, natural killer cells, neutrophils, T helper cells, Tfh cells, regulatory T cells, and tumor-infiltrating lymphocytes, was observed in the high risk cases in comparison with low risk ones. Among the 13 pathways, 12 except for MHC class-I pathway were found to be more active in high risk cases than in low risk ones (Fig. 8B). Moreover, a marked difference in the expression level of 35 immune checkpoints was observed between the 2 clusters of patients. Among the 35 checkpoints, CD200 and VTCN1 were up-regulated in low risk patients, and the remaining 33 were present at a higher level in high risk patients (Fig. 9).

## 4. Discussion

Here, we presented data showing that 32 out of 52 PRGs display differential expression between GBM versus normal tissue samples. Moreover, a PPI analysis revealed that *CASP1, GSDMD, NLRP1, AIM2, PYCARD, CASP8, CASP5, TP53*, and *CASP3* were hub genes. Recent researches have shown that activated *CASP1* could cleave C1q binding protein and boost aerobic glycolysis in tumor cells.^[14]^ Upon activation, *CASP3* can cleave GSDME to induce necrosis, providing novel insights into malignant tumor chemotherapy.^[15]^ *CASP8* has been defined as a molecular switch that regulates apoptosis, necroptosis, and pyroptosis; its activation participated in inflammatory responses of COVID-19 patients, probably causing lung injury.^[16,17]^ *NLRP1* is abundant in epithelial barrier tissues. And it could serve as a direct sensor for infection of dsRNA and RNA viruses, while being related to immune response.^[18,19]^ *AIM2* inflammasome contributes to surveillance of DNA damage and regulation of neurodevelopment.^[20]^ *PYCARD* is implicated in both pyroptosis and apoptosis.^[21]^

Pyroptosis plays a dual function in tumor progression and treatment. And it can lead to the release of inflammatory factors while transforming normal cells into malignant ones.^[22]^ Pyroptosis may potentially be used for prognostic prediction and treatment of malignant tumors.^[23]^ Herein, we divided a TCGA cohort of GBM cases into 2 groups based on the expression level of 52 PRGs and identified 32 DEGs between them. Further investigation revealed no marked differences in clinicopathologic characteristics between the 2 groups. Cox univariate analysis and LASSO analysis led to the development of a 9-gene signature (*GZMB, AREG, LOXL1, MSTN, PTX3, IGFBP6, STC1, POM121L9P, TGM2*) that was subsequently verified by external validation using a GEO dataset. *GZMB* was proven to function in pyroptosis of various cancers.^[24]^ *AREG* can diminish tumor resistance while averting immunosuppression induced by programmed cell death 1 ligand.^[25]^ *LOX* family is associated with glioma progression, and *LOXL1* can confer antiapoptotic activity and promote gliomagenesis through stabilizing BAG2.^[26]^ As a myogenesis inhibitor, *MSTN* regulates developmental maturation of skeletal myocytes and inhibits excessive cardiac autophagy to reduce cardiac hypertrophy.^[27]^ *PTX3* acts as a key player in humoral innate immunity that is implicated in resistance to certain pathogens as well as inflammation regulation, while it is associated with COVID-19, breast cancer, melanoma, and ischemia-reperfusion injury.^[28–31]^ A recent study shows that *IGFBP6*, an IGF-II inhibitor, is critically involved in suppressing cancer cell survival and migration, while regulating apoptosis and cell migration in glioma.^[32]^ *STC1* is a biomarker of cellular senescence, and the senescence-associated secretory phenotype has been identified as a promising therapeutic target for, and driver of, age related disorders ranging from neurodegenerative conditions to malignant tumor.^[33,34]^ *POM121L9P* is related to poor prognosis of patients with epithelial ovarian cancer.^[35]^ Besides, *TGM2* expression is correlated with development of colorectal cancer and endometrial cancer.^[36,37]^

The current study found that the DEGs were enriched in a number of pathways, including immune-associated pathways, chemokine signaling pathway, IL − 17 pathway, TNF pathway, NF − kappa B pathway, PI3K − Akt signaling, AGE − RAGE pathway, and so forth. It has been reported that both chemokines and the corresponding receptors regulate cell migration, affecting numerous biological and cellular processes as well as the pathogenesis of diseases including inflammatory disorders and malignant tumors.^[38]^ TNF signaling pathway is implicated in cancer metastasis, while participating in the occurrence of autoimmune and neuroinflammatory disorders.^[39,40]^ As the founding member of a new inflammatory cytokine family, IL-17 plays a host-protective role mainly due to its pro-inflammatory properties, and unrestrained IL-17 signaling contributes to immunopathology, autoimmune disorders, and tumor development.^[41]^ Increasing evidence shows that NF-κB signaling is critical for generating pro-inflammatory cytokine and chemokine cascades in response to acute respiratory virus infections.^[42]^ Recent studies demonstrated that AGE-RAGE signaling not only participates in chronic obstructive lung disease and diabetic kidney disease, but also regulates apoptotic signaling to promote tumor progression.^[43–45]^ PI3K − Akt signaling is implicated in regulating numerous cellular and physiological processes, such as cell division, differentiation, survival, and autophagy.^[46]^ Herein, we observed greater enrichment of the immune cell subpopulations and increased activities of the immune associated pathways in high risk GBM cases as compared to the low risk cases. Moreover, marked differences in the expression levels of the immune checkpoints were detected between the 2 groups of patients. In this case, CD200 and VTCN1 were highly expressed in low risk cases, and the remaining 32 checkpoints were present at a higher level in high risk cases. It has been reported that while CD200 can promote immunosuppression in tumor microenvironment,^[47]^ VTCN1 expression is related to reduced inflammatory CD4 + T-cell response within the microenvironment.^[48]^ Our findings indicate that the expression level of CD200 and VTCN1 may be relevant to a better OS of the low risk patients. To date, specific regulatory mechanism of CD200 and VTCN1 in GBM remains unclear. Besides, we presented a nomogram combining clinical features with the 9-gene signature which may have a clinical relevance for GBM. However, there are some limitations to our study, such as the fact that it has not been applied in clinical practice for a long period of time, and further exploration is needed on the mechanisms associated with the 9-gene signature in the development of GBM.

The current research identified certain PRGs that were differently expressed between GBM tumor versus normal tissues, indicative of an association of GBM with pyroptosis. Furthermore, we showed that the risk score calculated using the 9-gene signature potentially serves as an independent predictor for the OS of GBM patients. Further studies revealed that the DEGs between high risk versus low risk GBM cases were related to tumor immunity. Overall, we developed a new multigene signature for prognostic prediction of GBM patients, thereby laying a solid foundation of GBM immunotherapy.

## Acknowledgements

We appreciate the accessible data from the TCGA and GEO (GSE83300) network.

## Author contributions

**Conceptualization:** Xiao Jin.

**Data curation:** Xiang Zhao.

**Formal analysis:** Xiang Zhao.

**Visualization:** Xiang Zhao.

**Writing – original draft:** Xiao Jin.

**Writing – review & editing:** Xiao Jin.

## Supplementary Material










