# Supplementary material for: A new immune checkpoint-associated nine-gene signature for prognostic prediction of glioblastoma
Source: Medicine (Baltimore). 2023 Mar 3;102(9):e33150. doi: 10.1097/MD.0000000000033150 (PMC9981394; doi:10.1097/MD.0000000000033150)
Supplement: Supplementary file 1 [file medi-102-e33150-s001.pdf]

Table 1 We extracted 52 pyroptosis-related genes from prior papers[9, 11-13], and they are presented in Table 1.

BAK1
BAX
CASP1
CASP3
CASP4
CASP5
CHMP2A
CHMP2B
CHMP3
CHMP4A
CHMP4B
CHMP4C
CHMP6
CHMP7
CYCS
ELANE
GSDMD
GSDME
GZMB
HMGB1
IL18
IL1A
IL1B
IRF1
IRF2
TP53
TP63
AIM2
CASP6
CASP8
CASP9
GPX4
GSDMA
GSDMB
GSDMC
IL6
NLRC4
NLRP1
NLRP2

NLRP3
NLRP6
NLRP7
NOD1
NOD2
PJVK
PLCG1
PRKACA
PYCARD
SCAF11
TIRAP
TNF
GZMA
