# Supplementary material for: A new immune checkpoint-associated nine-gene signature for prognostic prediction of glioblastoma
Source: Medicine (Baltimore). 2023 Mar 3;102(9):e33150. doi: 10.1097/MD.0000000000033150 (PMC9981394; doi:10.1097/MD.0000000000033150)
Supplement: Supplementary file 2 [file medi-102-e33150-s002.pdf]

Table 2 According to the data in the TCGA cohort, we identified the 32 DEGs between normal and tumour tissues (Table 2).

gene	conMean	treatMean	logFC	pValue
BAK1	3.718151	9.636186	1.373877	0.000206
BAX	8.106617	25.97406	1.6799	0.000149
CASP1	0.788254	7.21394	3.194055	0.000144
CASP3	6.125076	21.96569	1.842452	0.000172
CASP4	0.435474	4.128241	3.244868	0.000144
CASP5	0.061156	0.400374	2.710778	0.000754
CHMP2A	36.14678	65.23708	0.851825	0.000779
CHMP4A	0.90498	1.456233	0.686284	0.003943
CHMP6	9.46136	13.93087	0.558166	0.004545
GSDMD	1.580061	8.166822	2.369795	0.000386
GSDME	2.775533	8.320441	1.583895	0.000214
GZMB	0.196755	0.771592	1.971435	0.01964
HMGB1	21.07411	44.49574	1.078195	0.000314
IL18	1.700836	6.964098	2.033692	0.00111
IRF1	1.64164	5.634814	1.77923	0.001146
IRF2	6.08482	12.8865	1.082574	0.001146
TP53	3.356874	23.0849	2.781759	0.000179
AIM2	0.105308	0.818265	2.957951	0.001719
CASP6	1.121892	6.636859	2.564567	0.00016
CASP8	0.621864	1.847415	1.570837	0.000779
CASP9	3.39541	5.047885	0.572093	0.022131
GSDMA	0.130446	1.065572	3.0301	0.017398
NLRC4	0.425578	1.136615	1.417247	0.001183
NLRP1	26.47746	20.35289	-0.37953	0.048516
NLRP2	0.626068	0.175402	-1.83565	0.001009
NLRP7	0.018756	0.005403	-1.79567	0.005199
NOD1	0.493013	1.418747	1.524918	0.001009
NOD2	0.117775	0.730806	2.63346	0.000386
PRKACA	25.60602	20.0382	-0.35373	0.017827
PYCARD	1.979503	8.175181	2.046112	0.00049
SCAF11	3.157605	4.875505	0.626721	0.003222
GZMA	0.222694	2.163158	3.280002	0.001473
