# Supplementary material for: A new immune checkpoint-associated nine-gene signature for prognostic prediction of glioblastoma
Source: Medicine (Baltimore). 2023 Mar 3;102(9):e33150. doi: 10.1097/MD.0000000000033150 (PMC9981394; doi:10.1097/MD.0000000000033150)
Supplement: Supplementary file 3 [file medi-102-e33150-s003.pdf]

Table 3 To assess the prognostic value of DEGs, we further employed Cox regression analysis to evaluate the correlations between each gene and survival status in the TCGA cohort. To prevent omissions,we set 0.2 as the cut-off P-value,and 20 survival-related genes were identified for further analysis.

id	HR	HR.95L	HR.95H	pvalue
TNFSF14	1.533474	1.205317	1.950975	0.000501
PLAUR	1.425348	1.196985	1.697278	6.94E-05
RETN	1.444181	1.162779	1.793686	0.000888
SOCS3	1.292007	1.112765	1.500121	0.000773
GZMB	1.75587	1.322386	2.33145	9.96E-05
C5orf46	1.60611	1.257377	2.051564	0.000148
TIMP1	1.252438	1.105187	1.419309	0.00042
AREG	1.491555	1.224107	1.817436	7.32E-05
BCL3	1.373575	1.140516	1.654259	0.00082
LOXL1	1.386075	1.183572	1.623225	5.09E-05
MSTN	0.777837	0.671787	0.900629	0.000781
PTX3	1.218589	1.087068	1.366022	0.000692
VDR	1.475924	1.179311	1.847138	0.000672
ADAMTS14	1.571883	1.241446	1.990273	0.000173
CD300E	1.796044	1.274319	2.53137	0.000824
IGFBP6	1.35478	1.171925	1.566167	4.05E-05
PODNL1	1.615044	1.296838	2.011328	1.86E-05
STC1	1.488309	1.209223	1.831807	0.000175
POM121L9P	1.716304	1.340391	2.197643	1.85E-05
TGM2	1.573747	1.239553	1.998043	0.000197

.
