# Supplementary material for: A new immune checkpoint-associated nine-gene signature for prognostic prediction of glioblastoma
Source: Medicine (Baltimore). 2023 Mar 3;102(9):e33150. doi: 10.1097/MD.0000000000033150 (PMC9981394; doi:10.1097/MD.0000000000033150)
Supplement: Supplementary file 4 [file medi-102-e33150-s004.pdf]

Table 4 Tumour classifification based on the pyroptosis-related DEGs. 169 GBM patients were grouped into two clusters according to the consensus clustering matrix (k = 2). There are 484 DEGs between the two clusters.

PTCH1	3.18486	1.37871	-1.20791	0.000321	0.00201
CXCL1	1.20467	5.969346	2.308935	1.17E-10	1.73E-08
P4HA2	2.270632	5.075812	1.160545	4.80E-08	2.18E-06
COL8A1	2.985139	7.828184	1.39088	4.36E-06	7.54E-05
B4GALT1	5.900339	14.49752	1.296937	2.93E-10	3.56E-08
GAL	1.467078	4.107173	1.485201	9.13E-05	0.000781
IL11	0.434875	1.771449	2.026258	8.47E-10	7.92E-08
CD274	1.346613	2.942568	1.12774	2.69E-05	0.000305
FPR1	15.20797	39.06314	1.36098	8.34E-11	1.34E-08
RNFT2	5.665471	2.593429	-1.12734	0.000136	0.001059
CDCP1	1.395966	3.338608	1.257982	3.08E-11	6.42E-09
IL21R	0.416865	1.42481	1.773118	2.72E-11	5.91E-09
CXCR2	0.546341	1.211821	1.149302	0.000149	0.001124
RAB38	0.741366	1.499194	1.01593	2.67E-06	5.22E-05
CEROX1	27.47324	10.92181	-1.33082	3.87E-05	0.000406
IL1RN	0.959711	2.738941	1.512946	2.37E-10	3.02E-08
CSPG5	57.57201	26.9398	-1.09563	1.79E-07	6.14E-06
TRIL	24.63424	12.31292	-1.00049	0.000304	0.001924
CD72	1.018891	2.136504	1.068253	3.46E-07	1.03E-05
VNN1	0.40438	0.817949	1.016299	6.22E-07	1.63E-05
RGS2	25.55457	62.55156	1.291465	3.42E-06	6.29E-05
GPC2	6.885468	3.278182	-1.07066	5.45E-05	0.000525
AL731567.1	0.391597	0.824071	1.073399	2.87E-08	1.47E-06
AL627309.6	0.727795	1.738508	1.256244	2.78E-08	1.44E-06
MS4A4E	0.386011	0.846837	1.133444	2.75E-07	8.71E-06
AC141557.2	0.829026	0.308988	-1.42387	0.000544	0.002995
PCDH15	1.035254	0.329899	-1.64989	0.000724	0.003706
GPR39	0.394652	0.880413	1.157599	1.29E-05	0.000172
CH25H	3.986685	10.68449	1.422256	1.78E-09	1.44E-07
IL6	0.606307	6.519243	3.426584	1.55E-22	2.52E-18
TNFSF14	0.327785	1.183702	1.852483	5.74E-10	5.99E-08
FAM20A	1.865603	5.233651	1.488176	1.36E-11	3.45E-09
HLA-DRB5	62.30547	125.2007	1.006812	0.00123	0.005558
MEX3A	9.390794	4.269201	-1.13728	5.99E-07	1.58E-05
GXYLT2	1.178338	2.409499	1.03198	2.69E-05	0.000305
PLAUR	5.285921	13.69243	1.373152	4.60E-13	2.58E-10
HP	2.773715	6.778947	1.289241	1.33E-07	4.87E-06

GPR84	1.283282	3.478236	1.438518	3.64E-14	4.68E-11
MARCHF9	48.76781	19.72783	-1.3057	0.000162	0.001193
AC124067.2	0.569865	1.208121	1.084073	1.09E-05	0.000151
CCL7	0.198605	2.546532	3.680558	1.33E-12	5.56E-10
SERPINE1	41.71838	157.5103	1.916691	8.63E-09	5.45E-07
AL355922.1	1.313148	3.846091	1.550363	7.74E-11	1.29E-08
CTSZ	57.48317	125.9256	1.13136	1.14E-10	1.70E-08
AC245128.3	0.452266	1.382913	1.612468	3.45E-10	4.10E-08
OSM	2.002569	5.689659	1.506491	6.67E-09	4.39E-07
LILRB2	1.098191	3.23417	1.558266	5.54E-13	2.91E-10
SLC26A10	1.836696	0.552773	-1.73235	0.001294	0.005765
IGDCC3	2.780012	1.210688	-1.19926	0.000287	0.001845
EMB	1.672908	3.72647	1.155451	1.45E-10	2.10E-08
RETN	0.432807	1.440617	1.734891	4.07E-11	8.08E-09
IDO1	0.486505	1.096714	1.172661	0.000453	0.002606
IGHV3-21	0.970532	2.494972	1.362176	0.000356	0.002181
KRT18	0.798313	1.842979	1.207013	2.25E-05	0.000264
NNMT	27.84579	73.78155	1.405801	3.91E-08	1.84E-06
CXCL5	0.874614	7.750096	3.147497	4.82E-12	1.50E-09
COL28A1	5.245374	2.100763	-1.32013	0.002072	0.008265
RNU6-1028P	1.311414	0.453594	-1.53165	0.000206	0.001434
PTGS2	1.314028	5.511976	2.068574	7.06E-10	7.00E-08
NABP1	0.533011	1.158294	1.119765	5.74E-10	5.99E-08
CLEC10A	0.468092	1.448519	1.629715	3.91E-08	1.84E-06
TRAPPC13P1	1.103878	0.522765	-1.07834	0.000207	0.001442
IGLV2-14	3.470055	10.60727	1.612022	0.000477	0.002717
RNU4-62P	0.599886	1.450271	1.273563	4.43E-06	7.63E-05
HAMP	4.007032	11.86564	1.566184	1.14E-09	1.00E-07
SOCS3	23.05483	68.46196	1.570234	7.48E-15	2.48E-11
LBP	0.545961	1.878461	1.782682	0.000335	0.002073
CXCL6	0.295729	2.754471	3.21943	5.05E-12	1.50E-09
KIF26A	1.375717	0.627177	-1.13324	0.001016	0.004803
TWIST2	0.47669	2.041317	2.098376	5.85E-06	9.31E-05
SIGLEC10	8.643494	17.46472	1.014757	1.53E-07	5.43E-06
AL121827.2	7.174104	3.583405	-1.00147	0.002311	0.00897
SFRP2	5.835453	18.85759	1.692229	0.000815	0.004061
CXCL2	2.735287	9.250832	1.757891	5.74E-10	5.99E-08
FAP	0.82988	1.85576	1.161035	0.000485	0.002746
IGLC2	6.157178	17.66201	1.520308	0.000188	0.001333
GZMB	0.487445	1.360253	1.480563	2.93E-08	1.48E-06
MMP19	1.75386	5.304114	1.596578	1.09E-09	9.62E-08
LYZ	20.19152	43.05675	1.09249	8.69E-07	2.16E-05
PCED1B-AS1	3.38015	7.174185	1.085728	1.81E-12	7.19E-10

VEGFC	0.668225	1.649569	1.303682	0.000196	0.001382
ASCL1	26.49991	11.73804	-1.1748	1.09E-07	4.21E-06
EFCAB1	0.414475	0.891102	1.104307	0.002367	0.009138
SPOCD1	8.347557	22.19682	1.410927	2.26E-10	2.93E-08
IL1R1	1.628796	5.804113	1.83327	1.19E-12	5.24E-10
CCER2	6.162336	3.08048	-1.00032	0.000479	0.002717
BTBD17	11.80157	4.444772	-1.4088	5.20E-08	2.31E-06
S100A8	11.99601	47.32696	1.980107	6.50E-13	3.31E-10
MIR222HG	0.538058	1.137855	1.080484	2.08E-06	4.32E-05
IL4R	3.799934	7.632524	1.006186	5.07E-12	1.50E-09
CCL8	0.840944	2.813969	1.742524	3.97E-10	4.61E-08
LOX	6.92725	18.9191	1.449489	7.05E-08	2.96E-06
SBK1	9.940714	4.764113	-1.06114	4.93E-06	8.19E-05
SIGLEC9	3.189261	6.845738	1.101984	3.00E-13	1.95E-10
COL5A1	6.707376	19.17563	1.515453	1.15E-06	2.68E-05
CTSS	16.91938	34.77298	1.03929	2.59E-08	1.35E-06
CXCL3	0.891922	4.547369	2.350043	1.22E-12	5.24E-10
AP006333.1	2.560268	1.134145	-1.17469	1.76E-05	0.000219
PI3	11.50183	81.94616	2.832813	3.40E-11	6.91E-09
GJB2	3.525572	10.90655	1.629265	4.44E-06	7.63E-05
PIK3C2B	4.795252	1.878529	-1.352	1.47E-05	0.00019
COL8A2	3.587228	7.641931	1.091068	6.37E-06	9.91E-05
C5orf46	0.12742	1.651878	3.696439	2.39E-08	1.26E-06
PTPRU	2.076273	4.482379	1.110268	0.000125	0.000992
LINC01605	0.340068	1.292063	1.92578	1.60E-08	8.94E-07
SLC11A1	5.512999	12.57485	1.189632	5.36E-10	5.77E-08
TREM1	1.99535	7.919012	1.988679	8.22E-12	2.33E-09
BATF	1.179514	3.094685	1.3916	8.82E-14	7.76E-11
TIMP1	198.4706	603.757	1.605043	2.18E-11	5.07E-09
CACNA2D4	0.570338	1.141684	1.001275	1.36E-11	3.45E-09
LINC02611	0.483205	0.96999	1.005333	1.26E-11	3.36E-09
FBLN2	2.124242	4.293088	1.015068	0.00094	0.004535
UBXN10-AS1	1.272653	3.5903	1.496265	2.04E-05	0.000245
S100A9	48.08546	175.3397	1.86648	1.48E-13	1.21E-10
TBX3	1.438395	0.642976	-1.16162	0.000395	0.002352
MS4A6A	12.13715	27.4762	1.178752	1.82E-09	1.46E-07
AC068643.1	0.819018	0.287321	-1.51123	1.37E-06	3.09E-05
EPHB1	9.267367	3.533944	-1.39088	7.17E-05	0.000645
FHL2	1.542176	3.56445	1.208712	3.60E-06	6.55E-05
RNASE3	1.332557	3.591254	1.43029	8.34E-11	1.34E-08
HK3	1.597729	4.97365	1.638282	5.99E-14	6.50E-11
FCGR2A	10.03269	25.25431	1.331821	9.06E-14	7.76E-11

PDZK1IP1	0.798614	2.788735	1.804041	1.07E-07	4.14E-06
HLA-DQB1	14.783	31.30669	1.082532	1.95E-05	0.000236
COL6A3	5.883092	13.82922	1.233074	1.09E-09	9.62E-08
INHBA	0.465	1.194416	1.361003	5.28E-05	0.000515
PPBP	0.667934	2.989897	2.162317	4.76E-06	8.00E-05
RHOD	2.134649	4.346375	1.025814	0.000165	0.001208
CDK4	222.8397	75.40365	-1.5633	0.000953	0.004583
SLA	5.041846	10.5234	1.061577	2.10E-08	1.12E-06
DCX	6.413097	3.119221	-1.03984	0.00066	0.003455
CD14	68.811	181.7706	1.401408	3.74E-14	4.68E-11
AC002401.4	0.266136	1.826883	2.779148	2.51E-06	4.98E-05
SNX20	0.827474	1.844058	1.156097	5.67E-11	1.00E-08
SOD2	52.84327	123.1945	1.221146	5.49E-09	3.72E-07
IGLC3	4.535686	10.96561	1.273593	3.08E-05	0.000341
UBD	0.89137	3.188952	1.838986	5.64E-08	2.48E-06
CD70	0.53865	4.476014	3.054794	1.74E-09	1.42E-07
GLIPR1	5.726411	11.6203	1.020944	6.90E-10	6.89E-08
PRR32	1.007279	0.486054	-1.05128	0.000151	0.001137
IGLV1-51	2.641337	8.750149	1.72804	0.000705	0.003632
AC008760.2	1.261913	3.019365	1.258633	6.37E-08	2.74E-06
MMD2	4.380586	2.127427	-1.04201	4.38E-05	0.000444
TDO2	0.790638	1.69936	1.103902	2.05E-07	6.88E-06
ALOX5AP	35.24877	74.90677	1.087523	4.90E-11	9.27E-09
RNASE1	67.02393	201.4545	1.587706	1.53E-06	3.38E-05
CXCL14	34.2049	88.88069	1.377667	7.34E-08	3.07E-06
LYVE1	2.109913	10.15088	2.266349	2.55E-09	1.92E-07
IGLV2-11	1.622812	5.380899	1.729351	0.00177	0.00732
CXCL13	1.129772	11.61706	3.362141	7.17E-05	0.000645
IL2RA	0.70285	4.821717	2.778258	2.35E-12	8.49E-10
COL15A1	1.165905	3.362703	1.528171	9.33E-08	3.74E-06
GLCCI1	8.93774	4.224468	-1.08114	4.68E-06	7.93E-05
NAMPTP1	2.681895	6.252354	1.221147	8.82E-09	5.50E-07
CRYBB2	0.627699	0.308977	-1.02257	0.000429	0.002498
IL24	0.303166	0.969284	1.676812	6.55E-05	0.000603
CXCL8	9.650629	71.73007	2.893883	3.00E-13	1.95E-10
SAA2	2.505153	9.276913	1.888746	1.78E-09	1.44E-07
RDH10	10.29065	22.50413	1.128856	7.59E-09	4.92E-07
CRYBG1	1.070737	2.576391	1.266747	3.75E-08	1.79E-06
NCF4	6.022019	12.30683	1.03114	6.71E-11	1.18E-08
PLIN2	11.1705	23.86307	1.095086	3.39E-07	1.01E-05
LILRB3	0.485738	1.202039	1.307235	1.29E-11	3.39E-09
F13A1	10.56514	68.19554	2.690365	7.27E-14	6.96E-11
TNFSF4	0.87257	2.025593	1.215001	1.85E-05	0.000227

LINC00460	0.223865	1.185505	2.404801	5.20E-05	0.000509
ROBO2	3.709529	1.808069	-1.03679	1.67E-08	9.26E-07
MMP7	2.199819	9.104723	2.04923	2.66E-09	1.97E-07
MFAP5	0.390839	1.472903	1.914015	0.002396	0.009214
HRH2	0.43369	0.949298	1.130197	8.45E-08	3.44E-06
S100A12	0.768351	2.685046	1.80511	2.49E-06	4.94E-05
TNFAIP2	6.802501	16.85076	1.308676	7.39E-10	7.20E-08
ACTG2	1.802114	8.224852	2.1903	5.04E-05	0.000497
IL10	0.578314	1.742224	1.591006	2.55E-09	1.92E-07
CSTA	2.014045	4.96452	1.301558	8.43E-12	2.33E-09
AREG	0.669929	3.205881	2.258641	7.49E-07	1.90E-05
LY96	14.91009	34.13303	1.194879	2.86E-10	3.50E-08
C1R	39.98595	85.53089	1.096952	4.13E-09	2.88E-07
AC124312.2	1.341764	0.620636	-1.11231	6.06E-05	0.000572
LINC00941	0.43357	1.25226	1.530196	3.02E-07	9.34E-06
LINC00645	1.456135	0.385077	-1.91892	0.000202	0.001411
SFN	0.838217	1.892306	1.17475	0.000196	0.001382
AQP9	1.233628	6.185656	2.326019	4.55E-11	8.82E-09
CNN1	1.171924	3.016696	1.364091	2.67E-06	5.22E-05
BCL3	5.696329	13.17656	1.20987	5.12E-13	2.78E-10
TNFSF9	0.561476	1.307764	1.219806	5.28E-05	0.000515
S100A11	179.4247	362.8712	1.016079	1.91E-12	7.22E-10
LRRC15	0.184867	1.194504	2.691854	2.24E-06	4.55E-05
TYMP	9.608128	20.65255	1.103992	1.74E-11	4.17E-09
THBS1	5.375289	24.79722	2.205764	1.16E-09	1.01E-07
C1S	25.35452	55.84326	1.13914	9.63E-11	1.51E-08
FCGR2B	1.161444	3.419102	1.557698	7.76E-11	1.29E-08
COL6A2	45.75739	137.3023	1.585279	5.85E-06	9.31E-05
SIGLEC7	1.687264	3.76626	1.158447	1.71E-10	2.35E-08
LAIR1	4.914307	10.52309	1.098499	1.50E-11	3.70E-09
MSC	1.441073	3.216363	1.158287	0.000435	0.002524
VSIG4	60.56337	150.1453	1.309842	1.36E-09	1.16E-07
ADAMTS1	4.653642	10.48531	1.171937	7.07E-05	0.000638
CHRDL2	0.71786	1.596744	1.153358	0.000753	0.003816
CD7	0.52163	1.05696	1.018822	6.29E-10	6.36E-08
KCNN4	1.247357	2.851334	1.192762	0.000669	0.003494
JAK3	1.288696	3.012989	1.225283	8.93E-13	4.27E-10
S100P	0.352719	0.848681	1.266701	0.001703	0.007089
NFKBIZ	1.72442	5.330531	1.628168	9.13E-15	2.48E-11
IGHV3-73	0.239882	1.018459	2.085994	0.000195	0.001379
ASPN	1.975823	4.645274	1.23331	0.000364	0.002213
ASIC4-AS1	1.489308	0.667978	-1.15677	0.000753	0.003816
SRPX2	10.74739	24.35236	1.180075	3.08E-06	5.86E-05

BCAN	420.0872	192.9451	-1.1225	1.21E-07	4.54E-06
NAMPT	34.82826	82.76294	1.248727	5.48E-10	5.87E-08
MCEMP1	0.216151	1.6266	2.91175	2.00E-13	1.48E-10
LINC01614	0.560638	2.535924	2.17737	0.000108	0.000888
PLA2G2A	29.42569	58.89824	1.001149	3.13E-06	5.95E-05
RCOR2	12.15732	5.321899	-1.19181	5.65E-06	9.09E-05
PDZRN4	1.52178	0.648027	-1.23163	0.000196	0.001382
AL392089.1	3.330052	1.664455	-1.0005	0.002202	0.008652
SLC16A10	0.504049	1.391696	1.465209	1.50E-08	8.45E-07
IGHA2	2.3377	5.587251	1.257049	9.55E-05	0.000809
GAPLINC	0.449425	0.932876	1.053605	3.80E-07	1.11E-05
PRF1	1.56351	4.920292	1.653956	1.52E-10	2.18E-08
HES5	4.902244	1.945209	-1.33352	6.75E-05	0.000618
PAEP	0.034305	1.542756	5.490933	0.000126	0.001001
FAM177B	0.621118	1.474606	1.24739	5.24E-10	5.68E-08
KCNB1	1.76823	0.87435	-1.01602	0.000326	0.002029
TGFBI	22.01895	66.91562	1.603597	2.01E-10	2.66E-08
CFB	0.581157	1.461749	1.330697	1.71E-09	1.41E-07
RHOH	0.354176	0.804677	1.183945	1.02E-12	4.60E-10
HCK	7.667471	15.35303	1.001701	5.74E-10	5.99E-08
SLAMF9	0.871979	1.791861	1.039093	0.001413	0.006162
PTGES	1.829579	6.746412	1.882608	2.60E-09	1.94E-07
SNX10	15.27772	31.14374	1.027513	1.86E-07	6.32E-06
AIF1	35.45027	75.19298	1.084801	6.44E-10	6.47E-08
PLA2R1	0.320965	0.867278	1.434078	2.24E-06	4.55E-05
EMILIN3	13.28704	5.351252	-1.31207	3.80E-06	6.79E-05
CD163	22.35086	67.11363	1.586275	1.79E-10	2.43E-08
CD209	0.454872	2.214059	2.28316	8.22E-06	0.00012
GRID2	2.281101	0.932009	-1.29131	0.001184	0.005409
LOXL1	3.816629	12.30197	1.688518	0.000724	0.003706
ZNF560	0.965963	0.102194	-3.24066	0.000965	0.004629
MYL9	19.38804	44.09576	1.185473	1.14E-08	6.84E-07
CCR2	0.312928	0.892049	1.511291	3.37E-10	4.03E-08
APCDD1L	0.415636	2.263078	2.444895	1.71E-06	3.67E-05
ARL11	0.887679	1.788271	1.010456	1.06E-09	9.61E-08
LINC02821	0.721377	2.013394	1.480805	9.84E-05	0.000829
AL627309.7	1.195727	2.743721	1.198246	8.92E-07	2.21E-05
LINC02293	0.732323	0.314307	-1.22031	7.49E-05	0.000668
ITGA3	8.660152	19.30871	1.156788	0.000123	0.000982
IGKV3-11	2.013324	5.453263	1.437541	0.002175	0.008589
CHD7	4.130494	2.046557	-1.01312	3.42E-06	6.29E-05
MMP1	0.717411	1.864895	1.378222	1.74E-09	1.42E-07
AL354919.2	4.009727	10.23156	1.35145	4.77E-10	5.29E-08

CTSB	147.8573	303.4237	1.037129	3.40E-11	6.91E-09
BX640514.2	0.550191	1.139478	1.05037	4.92E-06	8.19E-05
DLL3	36.3822	16.39841	-1.14968	0.000418	0.002451
VENTX	0.567232	1.497172	1.400229	2.41E-11	5.44E-09
STAB1	10.852	25.52679	1.234051	4.35E-12	1.45E-09
ICAM1	6.514013	19.79157	1.603267	1.47E-12	5.98E-10
MIR26A2	0.968734	0.453993	-1.09343	0.001983	0.007991
JCHAIN	3.248923	14.92537	2.199733	0.000108	0.000888
CAVIN3	3.635922	8.225981	1.177866	1.47E-07	5.29E-06
BIRC3	1.153187	3.471976	1.590131	2.13E-11	5.02E-09
MS4A7	8.51117	17.85333	1.068764	2.43E-08	1.27E-06
IGLV2-23	2.289641	4.659657	1.025102	0.000289	0.001856
DCC	1.187036	0.466909	-1.34615	0.000136	0.001059
SCIN	3.000265	6.680676	1.154904	1.15E-06	2.68E-05
SLPI	31.10856	66.97806	1.106377	1.67E-10	2.32E-08
LTF	63.11104	352.2589	2.480672	3.95E-07	1.14E-05
CELSR3	2.498663	1.243404	-1.00686	0.000763	0.003852
HIF3A	3.598612	1.537169	-1.22716	6.96E-05	0.000633
TNFRSF18	0.572198	1.499875	1.390256	3.54E-06	6.45E-05
CEACAM4	0.31438	1.020953	1.699336	5.87E-10	6.05E-08
CYP1B1	3.003305	7.667029	1.352117	4.41E-09	3.06E-07
NEU4	7.242262	3.374095	-1.10194	4.74E-05	0.000473
PLAU	12.08666	31.26265	1.371024	2.23E-09	1.72E-07
CADM2	9.887991	4.62271	-1.09694	3.24E-06	6.07E-05
THBD	2.62766	7.212219	1.456664	3.83E-12	1.33E-09
MSTN	7.567734	3.12915	-1.27409	0.001097	0.005098
PI15	0.436496	1.007708	1.207037	0.00019	0.001348
AC233296.1	0.810559	0.370115	-1.13094	0.000275	0.001788
CBX2	3.515408	1.725287	-1.02686	5.46E-06	8.86E-05
DOK2	1.155429	2.786277	1.26991	1.29E-08	7.55E-07
TLR8	0.770303	1.814467	1.236048	8.22E-07	2.06E-05
ABCC3	5.866257	11.96261	1.028021	2.22E-07	7.38E-06
HLA-DRB1	210.3591	423.0807	1.008079	8.27E-09	5.32E-07
IGLV4-60	0.190354	2.171375	3.51185	0.002647	0.009943
PTX3	11.53987	43.96133	1.929608	4.25E-10	4.81E-08
IGKV4-1	4.348356	14.41087	1.728615	0.00021	0.001459
GRIA4	7.159063	3.476849	-1.04199	2.08E-05	0.000248
B3GAT2	5.662494	2.522994	-1.1663	1.45E-05	0.000189
FCGR3B	0.778742	1.682154	1.111093	0.000364	0.002213
SMIM25	0.825569	2.145162	1.377626	9.93E-10	9.08E-08
MIR378B	0.874991	0.308953	-1.50188	0.000203	0.001418
P2RY6	0.840997	1.803116	1.100319	4.66E-10	5.24E-08
SLAMF8	2.658783	7.053941	1.407663	1.66E-11	4.03E-09

GFPT2	9.608134	20.36734	1.08393	3.14E-07	9.60E-06
SOX8	58.6673	24.38599	-1.2665	4.60E-07	1.28E-05
LINC00928	0.814319	0.383973	-1.08459	5.62E-05	0.000538
SAA1	39.5312	115.7099	1.549449	8.63E-09	5.45E-07
MS4A4A	8.693868	25.02193	1.525123	2.53E-11	5.56E-09
CHI3L1	779.6436	2353.991	1.594222	1.41E-08	8.13E-07
LINC01506	0.409819	0.855654	1.062041	7.97E-05	0.000701
IGLV3-1	1.298265	2.85056	1.134661	3.14E-05	0.000346
GALNT5	0.42364	1.377659	1.701308	6.83E-07	1.77E-05
AC106865.1	0.49914	1.292752	1.372928	1.44E-07	5.22E-06
IGKV1-16	0.635755	1.848897	1.540122	0.000598	0.003223
COL1A1	45.03022	126.0162	1.484644	1.29E-08	7.55E-07
DKK1	2.197304	9.432925	2.101971	4.07E-06	7.19E-05
CFI	11.46907	23.50623	1.035294	3.46E-08	1.69E-06
PCSK1	3.54336	7.12649	1.008074	2.52E-05	0.000289
CEMIP	2.184423	4.410841	1.013801	0.001056	0.004954
TNR	12.90919	4.485773	-1.52497	0.000158	0.00117
IGHG3	1.981774	8.765256	2.145004	0.000257	0.001698
VDR	0.410398	1.256711	1.614558	1.91E-12	7.22E-10
LIF	3.063205	12.80846	2.063983	5.07E-12	1.50E-09
CLCF1	3.377151	8.200405	1.279888	2.60E-09	1.94E-07
LINC01497	1.016484	0.47256	-1.10502	0.001702	0.007089
IL7R	0.518354	1.957115	1.916719	3.52E-13	2.20E-10
CA14	5.880558	2.842722	-1.04868	4.00E-06	7.09E-05
LINC01358	0.646401	1.315713	1.025343	0.000601	0.003224
IGLV3-21	2.667068	5.683761	1.091591	0.000175	0.001266
AC018755.4	1.595969	3.407145	1.094131	8.37E-07	2.09E-05
AL008726.1	0.611521	1.309106	1.098108	1.69E-07	5.86E-06
LILRA5	0.543061	2.211424	2.02579	8.43E-12	2.33E-09
ASIC4	10.79383	4.217514	-1.35574	0.00195	0.007882
TNFAIP8	0.611703	1.273937	1.058392	7.76E-11	1.29E-08
SEZ6	21.31204	7.55164	-1.49681	0.000142	0.001091
IL1A	0.651209	1.527672	1.230142	3.25E-09	2.36E-07
MS4A14	0.542172	1.288564	1.248943	6.77E-08	2.88E-06
BDKRB2	0.819282	2.051887	1.32452	5.87E-10	6.05E-08
ADGRE1	0.698052	2.043912	1.549926	1.16E-09	1.01E-07
IGKV3-20	6.810525	19.30397	1.50306	7.97E-05	0.000701
AC126407.1	1.139459	0.421209	-1.43574	2.04E-05	0.000245
SEZ6L	18.09902	7.551907	-1.261	1.74E-06	3.73E-05
ERRFI1	9.95072	20.66546	1.054349	5.62E-05	0.000538
RUNX2	0.679637	1.614217	1.247999	1.97E-08	1.07E-06
CCL13	0.079209	2.363847	4.899334	3.95E-13	2.30E-10
MAP3K8	1.897625	5.28542	1.477823	2.26E-14	4.09E-11

IGKV3-15	1.532437	4.379804	1.515039	0.000263	0.001728
VAMP8	33.39964	69.32704	1.053585	1.87E-09	1.49E-07
CCL18	3.17266	27.36235	3.108427	3.19E-06	6.02E-05
SPP1	1162.124	2603.902	1.163911	3.27E-07	9.88E-06
IGLV7-43	0.480971	1.371831	1.512081	0.002394	0.009214
DLL1	18.96074	4.932909	-1.94251	3.05E-05	0.000339
FOSL1	3.538993	9.820632	1.472477	1.27E-09	1.10E-07
MIR223HG	0.848601	1.977603	1.220594	2.09E-09	1.62E-07
C1QB	228.309	493.019	1.110656	7.91E-10	7.53E-08
SHISA6	2.812134	1.148028	-1.29251	0.001903	0.007736
OR2I1P	0.685703	2.551642	1.89577	3.91E-08	1.84E-06
NOD2	0.484498	1.158155	1.257264	2.55E-13	1.80E-10
IGKV1-5	4.238919	9.680755	1.191423	0.000143	0.001096
IGHG2	5.136981	17.78192	1.791419	0.000104	0.000867
MARCO	2.215051	8.607623	1.958275	1.31E-10	1.93E-08
CSF3	0.295282	2.413431	3.030921	2.60E-14	4.23E-11
IBSP	6.487714	24.13821	1.895536	1.71E-09	1.41E-07
FPR3	4.004322	8.779253	1.13254	4.26E-07	1.20E-05
CP	6.111144	15.85649	1.375559	1.72E-07	5.94E-06
MIR221	0.570272	1.356751	1.250434	1.70E-05	0.000213
VIPR2	3.779401	1.209551	-1.64369	0.001523	0.006506
HSD11B1	1.67797	3.681945	1.133751	1.53E-06	3.38E-05
ADAMTS14	1.072862	3.721265	1.794328	5.73E-09	3.85E-07
MAFB	17.97268	38.49138	1.09873	9.93E-10	9.08E-08
KCND2	7.816248	3.255532	-1.26358	7.63E-07	1.93E-05
CD300E	0.208153	1.191426	2.516974	2.29E-15	1.86E-11
RNF165	1.657642	0.789852	-1.06948	0.001746	0.007227
MYO1G	0.695831	1.898103	1.447749	6.69E-14	6.81E-11
LINC02513	0.428946	1.25655	1.550599	3.18E-08	1.59E-06
IGFBP3	51.01901	142.6172	1.483041	4.93E-06	8.19E-05
SPON1	24.69389	10.53994	-1.22829	0.000113	0.000918
EVA1A	0.886177	2.071283	1.224858	5.20E-08	2.31E-06
IER3	6.859187	22.62618	1.721883	8.14E-15	2.48E-11
TFPI2	1.151959	4.459866	1.952911	4.24E-08	1.97E-06
ADAMTS2	1.018329	2.296019	1.172931	5.56E-06	8.97E-05
IGLV6-57	0.7747	2.023384	1.385061	5.08E-05	0.0005
LRRTM1	1.481109	0.719189	-1.04224	0.000287	0.001845
TNFRSF10D	1.497457	3.003872	1.004309	3.60E-08	1.75E-06
C5AR1	8.052099	19.96911	1.310333	4.06E-10	4.62E-08
IGFBP6	7.97347	21.32285	1.419121	8.07E-07	2.03E-05
CHI3L2	70.52848	186.6892	1.40436	3.32E-08	1.64E-06
C15orf48	1.276151	3.52572	1.466119	4.89E-10	5.37E-08
FCGR3A	52.73894	116.109	1.138539	4.44E-11	8.71E-09

IGHM	2.085869	7.628953	1.870837	0.000652	0.003419
SP6	0.506227	1.12753	1.155309	0.000317	0.001991
MEDAG	0.649227	3.699176	2.510408	5.20E-08	2.31E-06
AC104695.3	0.6029	1.366547	1.180544	7.80E-08	3.21E-06
AC131097.2	3.492161	1.711672	-1.02871	0.000466	0.00266
AC061992.2	0.74136	1.841865	1.31292	3.97E-10	4.61E-08
TMEM150B	0.474391	1.170761	1.303297	1.60E-09	1.33E-07
TSHR	1.195485	0.237407	-2.33216	0.001703	0.007089
C6orf141	1.72841	3.653569	1.079861	1.59E-07	5.60E-06
MN1	2.240837	0.836708	-1.42124	0.000125	0.000992
CCL2	23.38859	88.6019	1.921533	3.64E-14	4.68E-11
COL1A2	47.41191	114.3623	1.27029	6.35E-05	0.00059
ANPEP	2.250826	7.070107	1.651278	3.59E-07	1.06E-05
MMP13	0.586487	6.207092	3.403747	0.000537	0.00296
CA12	10.53603	21.0908	1.001282	5.45E-05	0.000525
MXRA5	3.198609	8.469369	1.40481	6.33E-07	1.66E-05
VNN2	1.12633	2.734067	1.279419	1.15E-06	2.68E-05
C5AR2	0.41021	0.988395	1.268725	3.96E-09	2.78E-07
LRRC25	4.361631	9.11887	1.063987	5.02E-11	9.28E-09
ADAM8	1.847556	4.586752	1.311855	5.61E-09	3.79E-07
MNDA	5.939697	12.79954	1.10763	4.82E-09	3.29E-07
C1QA	168.0844	392.6356	1.224005	1.09E-10	1.64E-08
TNFRSF1B	11.28797	23.72134	1.071399	3.08E-11	6.42E-09
RARRES1	1.974019	16.79589	3.088901	3.25E-09	2.36E-07
LUM	11.73024	34.10491	1.539747	2.93E-08	1.48E-06
ADGRB1	13.70555	6.017966	-1.18741	8.73E-05	0.000752
DISP3	3.314806	1.290458	-1.36104	1.89E-08	1.04E-06
GCNT1	0.783659	2.001935	1.353096	3.11E-09	2.28E-07
GNA15	3.165901	7.291626	1.203625	1.48E-14	3.44E-11
FOLR2	15.3632	45.04689	1.551948	3.38E-08	1.67E-06
IL1B	2.992704	8.676246	1.535621	1.06E-10	1.61E-08
ZDHHC22	9.716523	4.389323	-1.14644	1.62E-06	3.53E-05
DPT	0.326683	2.792896	3.095797	0.000509	0.002844
CEBPB	14.54507	30.67867	1.076706	3.20E-12	1.13E-09
IGLV3-19	2.239695	5.807215	1.374544	0.000834	0.004141
MYT1	4.063784	1.690973	-1.26497	0.001523	0.006506
KRT17	0.437522	2.623707	2.584179	1.30E-06	2.97E-05
LRRN2	21.44567	6.080453	-1.81844	0.000165	0.001208
MYCN	6.300553	2.87023	-1.13431	4.26E-07	1.20E-05
CCL20	0.676378	4.908111	2.859265	9.66E-13	4.49E-10
CCL26	0.412085	1.934471	2.230925	2.44E-06	4.88E-05
CTSC	5.92368	14.961	1.33664	2.00E-13	1.48E-10
PODNL1	0.800407	3.01114	1.911503	4.96E-07	1.35E-05

MME	0.211019	1.176976	2.47964	0.000101	0.000849
IL1R2	0.637611	2.231951	1.807556	7.86E-05	0.000693
SERPINA1	11.33634	32.64014	1.525693	4.67E-14	5.43E-11
KRT8	0.22137	1.042375	2.235341	8.22E-06	0.00012
SRGN	93.68069	221.9828	1.244624	7.39E-10	7.20E-08
PTPN22	0.369481	0.765215	1.050366	1.07E-08	6.52E-07
ANGPTL4	19.8536	44.13522	1.152529	7.43E-06	0.000112
MSLN	0.741704	1.925026	1.375963	0.002311	0.00897
DCBLD2	3.829376	8.83705	1.206456	4.78E-07	1.32E-05
COL11A2	2.045035	0.945988	-1.11223	0.000147	0.001112
SAPCD2	9.095854	4.454228	-1.03003	8.73E-05	0.000752
FCGBP	26.39786	62.09733	1.23411	6.15E-10	6.30E-08
SALL3	7.40738	3.394766	-1.12565	1.42E-07	5.14E-06
MRC1	1.458665	7.488629	2.360053	2.14E-08	1.14E-06
STC1	2.627357	9.701495	1.884595	5.40E-11	9.76E-09
IGLV10-54	0.821837	4.096417	2.317438	0.000655	0.003438
ALOX5	5.566133	12.01736	1.110373	9.18E-11	1.47E-08
TREML3P	0.370805	1.157509	1.642289	1.93E-08	1.05E-06
AL133371.2	1.097911	2.43704	1.150369	3.47E-09	2.50E-07
BCL2A1	4.22445	12.94654	1.615731	3.71E-13	2.24E-10
RNASE2	8.649615	25.7165	1.571987	4.46E-15	2.42E-11
CST7	1.450303	3.054721	1.074686	6.91E-08	2.92E-06
CCN1	38.10228	90.37635	1.246068	8.22E-06	0.00012
HSPA7	2.703053	5.534197	1.033784	9.71E-07	2.37E-05
IGHV3-33	1.02495	2.369996	1.209332	0.00022	0.001507
S100A4	25.3724	59.06802	1.219117	4.06E-10	4.62E-08
CALHM6	4.973613	10.45159	1.071356	8.27E-09	5.32E-07
POM121L9P	0.616174	1.346869	1.128199	0.000555	0.003037
ARSI	1.940415	5.656549	1.543557	0.000207	0.001442
RPRM	8.782331	3.156863	-1.47611	0.000977	0.004682
FPR2	0.312557	1.776227	2.506624	2.26E-14	4.09E-11
IGKC	13.28075	30.23948	1.187097	0.000116	0.00094
AC073349.2	0.731251	0.292203	-1.3234	5.36E-05	0.000521
TGM2	5.785001	13.14875	1.184537	1.09E-09	9.62E-08
LTBP2	2.187738	5.397614	1.302882	1.53E-09	1.29E-07
C1QC	232.1311	473.1787	1.027445	1.74E-09	1.42E-07
TLR2	5.617644	11.3622	1.016205	2.37E-10	3.02E-08
CLDN23	0.762968	1.883807	1.303957	4.78E-11	9.15E-09
ZC3H12A	1.358896	3.487927	1.359934	2.35E-12	8.49E-10
GZMA	1.535975	3.440398	1.163421	1.33E-07	4.87E-06
SOX4	37.66814	17.30174	-1.12243	2.53E-06	5.00E-05
FREM1	1.181732	0.478578	-1.30408	0.001974	0.007954
TNFSF10	3.834839	8.127269	1.083605	4.03E-07	1.15E-05

IGHV4-31	0.463789	0.996253	1.103044	7.03E-05	0.000638
TNFRSF13C	0.977679	0.441265	-1.14772	0.00018	0.001293
LMO1	7.793268	3.535585	-1.14028	0.000279	0.001806
THSD4	0.667342	0.279353	-1.25634	0.001581	0.006709
IGLV4-69	0.677091	1.913516	1.498804	0.002498	0.009533
SAA2-SAA4	1.568228	4.087377	1.38204	1.85E-07	6.32E-06
MYBPH	3.336578	7.763463	1.218331	7.35E-07	1.88E-05
TNFAIP3	3.325154	7.558567	1.184692	2.06E-10	2.71E-08
FCER1G	62.8495	133.1313	1.082877	8.69E-13	4.27E-10
EPYC	0.023108	1.759251	6.250429	0.000987	0.004726
IGKV3D-20	0.272618	1.048507	1.943384	0.000849	0.004193
CSF2RB	1.007139	2.25767	1.164572	3.62E-09	2.59E-07
COL6A1	80.67498	171.9901	1.092132	0.000678	0.00353
AC093895.2	0.367546	1.373399	1.901753	1.59E-10	2.25E-08
RNA5SP508	1.076076	0.531688	-1.01713	0.000518	0.002885
