# Supplementary material for: A new immune checkpoint-associated nine-gene signature for prognostic prediction of glioblastoma
Source: Medicine (Baltimore). 2023 Mar 3;102(9):e33150. doi: 10.1097/MD.0000000000033150 (PMC9981394; doi:10.1097/MD.0000000000033150)
Supplement: Supplementary file 5 [file medi-102-e33150-s005.pdf]

Table 5 We utilized the“limma” R package to extract the DEGs by applying the criteria FDR < 0.05 and |log2FC | ≥ 1. In total, 97 DEGs between the low- and high-risk groups in the TCGA cohort were identified. Among them, 90 genes were upregulated in the high-risk group, while the other 7 genes were downregulated (the data are shown in Table 5).

SERPINA3	4.161289	5.18627	1.024981	9.04E-09	6.15E-07
SRPX2	4.372128	5.439314	1.067186	1.48E-05	0.000179
BCAN	9.466902	8.190726	-1.27618	1.06E-05	0.000139
NAMPT	6.165058	7.32683	1.161772	1.45E-07	5.18E-06
PLA2G2A	4.018125	5.078303	1.060179	3.33E-05	0.000338
HES5	4.529206	3.362102	-1.1671	4.30E-07	1.15E-05
TGFBI	6.202425	7.377647	1.175223	1.87E-08	1.07E-06
CD163	5.725018	7.290629	1.565611	4.56E-10	6.88E-08
LOXL1	3.824279	5.014827	1.190548	5.56E-10	7.87E-08
ITGA3	4.0078	5.04093	1.03313	8.23E-08	3.31E-06
ICAM1	4.13801	5.295193	1.157182	2.93E-10	4.78E-08
BIRC3	2.576575	3.643946	1.067372	2.03E-09	2.05E-07
SLPI	4.940697	6.140301	1.199604	2.64E-06	4.76E-05
LTF	5.151051	7.226698	2.075647	8.91E-06	0.000121
CYP1B1	3.33723	4.5791	1.24187	9.78E-10	1.19E-07
PLAU	4.999953	6.347786	1.347833	1.08E-11	5.01E-09
THBD	3.547356	4.667712	1.120356	5.61E-11	1.56E-08
C1RL	3.683182	4.825767	1.142585	6.93E-13	6.41E-10
MSTN	3.915921	2.349608	-1.56631	2.85E-13	4.94E-10
ABCC3	3.444199	5.019509	1.57531	1.83E-11	7.24E-09
PTX3	4.524847	6.262357	1.73751	2.24E-10	3.83E-08
SAA1	3.775216	5.438365	1.663149	5.22E-06	8.09E-05
CHI3L1	8.66809	11.12175	2.453658	9.84E-12	4.82E-09
COL1A1	5.033718	6.896568	1.86285	8.41E-10	1.06E-07
CFI	4.458215	5.937402	1.479187	1.17E-12	8.56E-10
LIF	2.941208	4.430298	1.48909	8.29E-15	5.31E-11
HES6	7.626252	6.285976	-1.34028	5.09E-09	3.99E-07
CLCF1	3.144415	4.167079	1.022663	4.35E-11	1.37E-08
VASN	4.611853	5.654997	1.043144	8.00E-09	5.72E-07
CCL18	1.999655	3.218896	1.219241	0.000165	0.001188
SPP1	10.92698	12.11136	1.184376	1.54E-07	5.40E-06
MARCO	2.55319	3.900638	1.347448	9.04E-09	6.15E-07
IBSP	3.168439	4.477005	1.308566	9.34E-06	0.000126
FAM20C	6.770482	7.932949	1.162467	4.99E-13	5.77E-10
IER3	4.504888	5.59495	1.090062	5.09E-10	7.43E-08

OLIG2	6.876922	5.815665	-1.06126	3.00E-05	0.000314
C5AR1	3.717602	4.751412	1.03381	9.08E-11	2.13E-08
IGFBP6	3.714459	4.995097	1.280638	2.90E-09	2.61E-07
ANXA2	6.340774	7.444503	1.103729	2.78E-08	1.44E-06
CHI3L2	6.380529	8.009828	1.629299	4.53E-08	2.14E-06
CCL2	5.856367	7.439892	1.583525	2.67E-09	2.50E-07
COL1A2	5.229372	6.418405	1.189033	9.57E-07	2.19E-05
ANPEP	2.391904	3.469261	1.077358	1.57E-09	1.73E-07
LUM	3.389803	4.61301	1.223207	5.33E-07	1.38E-05
CCL20	1.847522	2.955659	1.108137	3.50E-10	5.39E-08
SERPINA1	4.934848	5.996877	1.062029	5.68E-10	7.96E-08
ANGPTL4	5.324445	6.650535	1.32609	8.55E-08	3.37E-06
FCGBP	6.390387	7.515865	1.125478	8.76E-07	2.05E-05
BCL2A1	3.835127	4.894466	1.059339	2.56E-09	2.44E-07
RNASE2	4.014093	5.114746	1.100653	6.77E-10	9.20E-08
MYBPH	2.927146	3.980707	1.053561	2.15E-08	1.17E-06
COL6A1	6.720332	7.839805	1.119474	3.00E-10	4.83E-08
